# Immune Activity, Body Condition and Human-Associated Environmental Impacts in a Wild Marine Mammal

**DOI:** 10.1371/journal.pone.0067132

**Published:** 2013-06-28

**Authors:** Patrick M. Brock, Ailsa J. Hall, Simon J. Goodman, Marilyn Cruz, Karina Acevedo-Whitehouse

**Affiliations:** 1 Institute of Zoology, Zoological Society of London, Regents Park, London, United Kingdom; 2 Institute of Integrative and Comparative Biology, University of Leeds, Leeds, United Kingdom; 3 Sea Mammal Research Unit, University of St. Andrews, Fife, United Kingdom; 4 Galapagos Genetics, Epidemiology and Pathology Laboratory, Galapagos National Park, Puerto Ayora, Ecuador; 5 Unit for Basic and Applied Microbiology, Autonomous University of Queretaro, Queretaro, Mexico; Swedish University of Agricultural Sciences, Sweden

## Abstract

Within individuals, immunity may compete with other life history traits for resources, such as energy and protein, and the damage caused by immunopathology can sometimes outweigh the protective benefits that immune responses confer. However, our understanding of the costs of immunity in the wild and how they relate to the myriad energetic demands on free-ranging organisms is limited. The endangered Galapagos sea lion (*Zalophus wollebaeki*) is threatened simultaneously by disease from domestic animals and rapid changes in food availability driven by unpredictable environmental variation. We made use of this unique ecology to investigate the relationship between changes in immune activity and changes in body condition. We found that during the first three months of life, changes in antibody concentration were negatively correlated with changes in mass per unit length, skinfold thickness and serum albumin concentration, but only in a sea lion colony exposed to anthropogenic environmental impacts. It has previously been shown that changes in antibody concentration during early Galapagos sea lion development were higher in a colony exposed to anthropogenic environmental impacts than in a control colony. This study allows for the possibility that these relatively large changes in antibody concentration are associated with negative impacts on fitness through an effect on body condition. Our findings suggest that energy availability and the degree of plasticity in immune investment may influence disease risk in natural populations synergistically, through a trade-off between investment in immunity and resistance to starvation. The relative benefits of such investments may change quickly and unpredictably, which allows for the possibility that individuals fine-tune their investment strategies in response to changes in environmental conditions. In addition, our results suggest that anthropogenic environmental impacts may impose subtle energetic costs on individuals, which could contribute to population declines, especially in times of energy shortage.

## Introduction

Maintaining the immune system and mounting immune responses are costly activities. The cost of immunity can be evolutionary or genetic, if immune function is selected for and covaries negatively with other fitness-enhancing traits [1]. The cost of immunity can also be energetic or physiological, if an immune response consumes resources such as energy and protein that consequently cannot be invested in other activities such as growth, or causes immunopathology [2]–[5]. Due to such inherent physiological costs, maximal immune responses are unlikely to be optimal, and investment in immunity must be balanced to maximise fitness [6]–[7]. The discipline of ecological immunology or wild immunology aims to disentangle how organisms manage this allocation problem in a variable environment, and to define immunity as a life history trait, theoretically and empirically [8]–[10].

In both vertebrates and invertebrates, an experimental increase in energy expenditure on immunity can decrease investment in other life history traits [11]–[14]. Complementarily, an experimental increase in energy expenditure on activities such as rearing, begging and sexual behaviour can decrease immune activity [15]–[18]. However, there are relatively few studies relevant to the costs of immunity in wild mammals (but see [19]–[20]), so in this study we investigated whether observable patterns were consistent with a physiological cost of immunity in the endangered Galapagos sea lion, by testing for correlations between changes in immune measures with changes in body condition in known individuals over time.

Given the complexity of immune dynamics in natural populations (e.g. [20]), a physiological cost associated with immunity may only be observable under certain ecological conditions [21]–[22]. The ecological circumstances of the Galapagos sea lion are defined by a combination of food limitation, disease threat and distinct colony differences in human impact, which make it a suitable system in which to investigate the relationship between energy availability and immunity, and one that could provide insight into the physiological costs of immunity in the wild. We tested two hypotheses: 1) that increases in immune measures over time were negatively correlated with decreases in body condition, and 2) that any such negative correlations were more pronounced in a human-impacted colony than in a comparison colony on an uninhabited island. Correlational evidence consistent with a physiological cost of maintaining immune protection [23] or mounting immune responses [4] in this system could have important ramifications for Galapagos sea lion conservation, and wider implications for the role of immune variation in the dynamics of wild populations.

## Methods

### Study System and Sampling

The Galapagos sea lion (*Zalophus wollebaeki*) is a useful system in which to investigate the relationship between energy availability and immunity for two reasons. First, the species is sensitive to changes in ocean productivity [24]–[25], so its small population (20,000–40,000 animals) undergoes stochastic decreases in size due to food limitation [26]. Second, there is a single Galapagos sea lion colony located in the centre of a rapidly growing town (Puerto Bazquerizo Moreno, San Cristobal). Due to the geographical isolation of the Galapagos archipelago and the spatial aggregation of pinnipeds into colonies, the comparison of this unique colony with those located in the protected zone of the Galapagos National Park provides an opportunity akin to a microcosmic natural experiment on the effects of anthropogenic influence on immune system development and activity in a wild mammal. The sea lions resident in the human-impacted colony of Puerto Bazquerizo Moreno on San Cristobal are exposed to two influences associated with humans that are relevant to immunity: domestic animals [27] and pollution [28]. Disease from domestic animals is a substantial threat to wild carnivores [29]–[31], including pinnipeds [32], so Galapagos sea lion immunity may play an important role in protection against emergent pathogens [33]. Despite the agriculture and tourism on San Cristobal, there is no evidence that the levels of chemical pollutants such as polychlorinated biphenyls (PCBs), polybrominated diphenyl ethers (PBDs), dichlorodiphenyltrichloroethane (DDT) and hydrocarbons are present in the bay at higher than background levels [28], [34]. However, sewage from the town water system, which is contaminated with faecal coliform bacteria [35], is deposited in the bay, and higher than background concentrations of faecal coliform bacteria have been recorded there [36]. Although there are other human settlements on the Galapagos Islands of Santa Cruz (Puerto Ayora), Isabela (Puerto Villamil) and Floreana (Puerto Velasco Ibarra), and a small number of juvenile and adult Galapagos sea lions visit these towns from nearby colonies, none are home to a breeding colony like Puerto Bazquerizo Moreno on San Cristobal.

We collected data in two Galapagos sea lion colonies, one in the town of Puerto Bazquerizo Moreno, San Cristobal (human-impacted colony; 0°54′07″ S, 89°36′44″ W) and the other in Bahia Paraiso, on the island of Santa Fe, where there are no resident humans or domestic animals (control colony; 0°48′15″ S, 90°02′28″ W). We sampled 30 juveniles in each colony at 6 months of age during April 2009, marked them with the tagging method of a long-running Galapagos sea lion study [37], and re-sampled them at 12, 18 and 24 months of age. We sampled 30 pups in each colony shortly after birth during November 2009, marked them by shaving, and re-sampled them 2 months later. All work was carried out under Galapagos National Park permits PC-18-09, N°046-2009-PNG, N°101-2010-PNG and N°032-2010-PNG. Samples were imported to the UK under DEFRA permits POAO/2008/925 and POAO/2010/136.

### Quantifying Immune Activity

Quantifying immune system development and activity in the wild is a challenge, especially in a species for which no laboratory reagents have been specifically developed [10]. We have previously measured variation in 12 immune-related physiological measures during the first two years of life in the Galapagos sea lion [38], and discussed its significance in the conceptual framework of ecological immunology [8]–[10]. We found that Galapagos sea lions from the human-impacted colony on San Cristobal had relatively higher levels of immune activity – quantified using cell-mediated and humoral immune components, and snapshot and cumulative measures – than sea lions from a colony on the uninhabited island of Santa Fe [38]. In this study we used three of the previously described immune measures: total immunoglobulin G (IgG) concentration, the *in vivo* inflammation response to phytohemagglutinin (PHA), and total leukocyte concentration. To aid the interpretation of this immune variation we took repeated measurements of known individuals from two age classes [38]: pups (3 months or younger) and juveniles (6 months or older). In pups, in which the involution of the thymus is unlikely to have taken place [39], increases in IgG and total leukocyte concentrations are likely to be driven by the establishment of protective baseline levels in response to the post-natal antigenic environment [40]. In juveniles, in which immune systems are likely to have matured, changes in immune measures are likely to represent responses to infection. In addition, we have shown that previous exposure to PHA does not have an effect on the magnitude of the induced swelling in Galapagos sea lions of any of the ages included in this study [38].

We took a 7.5 ml blood sample from the caudal gluteal vein of each individual during each capture [41]. We allowed 6 ml of blood to clot and then centrifuged it at 3000 rpm for 15 minutes to extract serum, which we stored at −80°C. To determine albumin concentration we carried out serum protein electrophoresis using SAS-MX serum protein kits (Helena Biosciences, UK), and report albumin concentration as the ratio of albumin peak intensity to the total peak intensity of the albumin, alpha globulin and beta globulin protein fractions, as described previously [38].

We measured IgG concentrations in serum with a protein A ELISA as reported previously [19], [38]. We calculated the PHA response as the difference between the change in median thickness of the right hind flipper-webbing induced by a 0.05 ml intra-dermal injection of phosphate buffered saline (P3813, Sigma-Aldridge, UK) and the change in median thickness of the left hind flipper-webbing induced by a 0.05 ml intra-dermal injection of 100 µg/µl PHA solution (L8754, Sigma-Aldridge, UK). We took all flipper-webbing measurements three times to the nearest 0.01 mm using a thickness gauge (7/7309, Mitutoyo, UK), and excluded sets of repeated measurements with coefficients of variation greater than 25%. We measured total leukocyte concentration using a haemocytometer (Neubauer, Philip Harris, UK) after diluting 20 µl of blood in 380 µl of Rees-Ecker solution (sodium citrate 3.8 g, formalin 40% 0.2 ml, brilliant cresyl blue 0.1 g; Fisher Scientific, UK). For graphical and statistical summaries of the variation in these immune measures with colony and age, see Brock et al. 2012 [38], which also includes detailed descriptions of the data collection and laboratory protocols.

### Quantifying Body Condition

We used three measures of body condition to assess nutritional status: mass per unit length, serum albumin concentration and skinfold thickness. We do not consider these to be indicators of the underlying and immeasurable ‘quality’ of an individual, but rather as different aspects of dynamic physiological state associated with resource availability [42]. The relationship between body mass and body length is used as a measure of condition in many vertebrate taxa, and there are a number of ways in which it can be expressed [19], [43]–[44]. Variation in mass per unit length is most often calculated for adults, and it is assumed that higher values are indicative of better nutritional condition. If measured in immature animals at multiple time points, mass per unit length can also serve as an indicator of relative investment in the growth of different tissues, as it describes how skeletal size changes with overall tissue mass [45]–[46]. Despite debate over the calculation and interpretation of mass per unit length [47], this measure has been correlated with fitness-related traits in many species, including pinnipeds [19], [48]–[49]. Albumin is a transporter molecule and protein reservoir, and its concentration in serum is commonly used to diagnose malnutrition in marine mammals [41]. Skinfold thickness is a measure of how much fat is stored under mammalian skin [50]–[51] and is likely to be better correlated with total body fat in pinnipeds than in other mammalian taxa, as the majority of pinniped fat is stored subcutaneously and relatively little is stored in internal deposits [52]. Total body fat is an important determinant of fitness in marine mammals as it is correlated with their ability to resist starvation [53].

We measured body mass to the nearest 0.5 kg using a spring balance (Pesola, Switzerland) and curved body length to the nearest 0.5 cm with a tape measure. We re-measured body length 24 hours after initial capture during re-captures and used the average of the two values for analysis. In pups, we took three repeated measurements of dorsal axial skinfold thickness [50] to the nearest 0.01 mm with callipers (Wiha, USA). We calculated mass per unit length as the residuals of a linear regression between body mass and mean body length. In juveniles, mass and length were log-transformed prior to regression to normalise residuals (Shapiro-Wilk, p>0.05). We calculated pup skinfold thickness as the median of the three repeated measures, and excluded sets of repeated measurements with coefficients of variation greater than 25%. We did not measure skinfold thickness in juveniles, as it was not possible to safely remove their heads from nets during capture.

### Statistical Analysis

Prior to analysis, we calculated absolute changes in immune and condition variables between consecutive time points for each individual. Correlations amongst changes in immune and condition variables were non-significant in both pups and juveniles (p>0.05). First, we tested for colony and sex differences in condition changes by fitting analysis of variance models (ANOVAs) to pup data, and linear mixed effect (LME) models that included period of change and individual identity as random effects to juvenile data. Next, we fitted the nine possible linear models to test the effect of change in a single immune variable on change in a single condition variable in pups. We fitted change in condition as the response and change in the immune measure, sex, colony and their interactions as explanatory terms. In juveniles, we could fit six such models, as we did not collect data on juvenile skinfold thickness; we fitted these as LME models including individual identity and period of change as random effects. As the first step in model selection we compared these 15 maximal models with null models, using F-tests for pup data and likelihood ratio tests for juvenile data. We further considered only those relationships for which maximal models performed significantly better than null models.

In order to control for possible sex differences and to avoid over-complicating models, we split data for further analysis by colony. For each relationship selected by the null model comparison, and for each colony, we fitted change in condition as the response and change in the immune measure, sex and their interaction as explanatory terms. Then we compared these models to models without the interaction using F-tests in pups and likelihood ratio tests in juveniles. As before we fitted linear models to pup data and LME models that included period of change and individual identity as random effects to juvenile data. We checked all models for signs of heteroscedasticity, heterogeneity of variance, non-normality of error and the disproportionate influence of outliers [54], and carried out all analyses in R 2.11.1 [55].

## Results

In pups, change in mass per unit length was higher in the control colony than in the human-impacted colony (contrast estimate = 0.85 kg, SE = 0.41 kg, *t*_2,52_ = 2.08, *p* = 0.042), and there was no sex difference (*t*_2,52_ = 1.52, *p* = 0.132). Change in skinfold thickness was higher in males than in females (contrast estimate = 0.094 cm, SE = 0.045 cm, *t*_2,52_ = 2.07, *p* = 0.042), and there was no difference between colonies (*t*_2,52_ = −0.16, *p* = 0.873). There was neither a colony difference (*t*_2,39_ = −1.34, *p* = 0.185) nor a sex difference (*t*_2,39_ = −1.56, *p* = 0.125) in change in albumin concentration.

In juveniles there were neither colony differences (N_total_ = 73, N_individuals_ = 38, *t*_68_ = 1.11, *p* = 0.271) nor sex differences (N_total_ = 73, N_individuals_ = 38, *t*_68_ = −0.85, *p* = 0.396) in change in mass per unit length or albumin concentration (colony, N_total_ = 60, N_individuals_ = 36, *t*_55_ = −0.86, *p* = 0.389; sex, N_total_ = 60, N_individuals_ = 36, *t*_55_ = 0.26, *p* = 0.792). Table S1 shows mean changes in all immune and condition variables by age class and colony.

**Figure 1:**
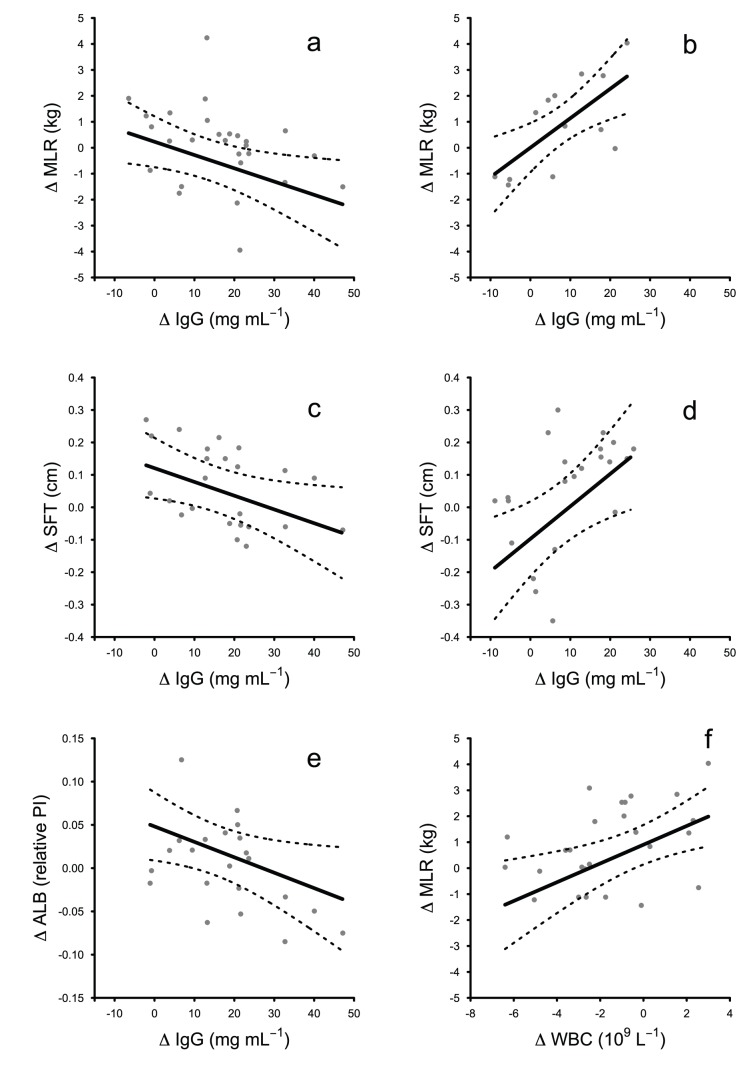
Predicted relationships between changes in immune measures and changes in body condition in Galapagos sea lion pups resident in the human-impacted (A, C, E) and the control (B, D, F) colonies. Dotted lines represent 95% confidence intervals, ‘Δ’ denotes ‘change in’, ‘MLR’ mass per unit length (kg), ‘SFT’ skinfold thickness (cm), ‘ALB’ albumin concentration (relative peak intensity), ‘IgG’ total immunoglobulin G concentration (mg mL^−1^), ‘WBC’ total leukocyte concentration (10^9^ L^−1^) and ‘PHA’ response to phytohemagglutinin (mm). Note that the relationship shown in (B) is for females only.

Six of the 15 maximal models of the effect of change in a single immune variable on change in a single condition variable explained significantly more variation than equivalent null models (Table 1). In pups, there was a negative relationship between changes in all 3 measures of condition and changes in IgG concentration in the human-impacted colony (Table 2; Fig. 1A, C, E; Table S2). In the control colony there was a positive relationship between change in skinfold thickness and change in IgG concentration, and between change in mass per unit length and change in total leukocyte concentration (Table 2; Fig. 1D, F; Table S2). In addition, there was a positive relationship between change in mass per unit length and change in IgG concentration in females of the control colony (Table 2; Fig. 1B; Table S2). In juvenile males from the human-impacted colony, changes in albumin concentration were negatively related to changes in IgG concentration (Table 2; Table S3). For further context of these results see Brock et al. 2012 [38], which summarises variation in these immune measures with age and colony.

**Table 1:** Comparison of full and null models on the effect of changes in immune measures on changes in body condition; F-tests in pups, likelihood ratio tests in juveniles.

		Pups			Juveniles			
Condition Variable	Immune Variable	N	F	*p*	N_total_	N_individuals_	Likelihood Ratio	*p*
ΔMLR	ΔIgG	51	3.452	0.005**	73	38	4.991	0.661
ΔMLR	ΔPHA	55	1.205	0.319	61	36	12.860	0.075
ΔMLR	ΔWBC	51	2.431	0.034*	84	45	2.687	0.912
ΔSFT	ΔIgG	47	2.578	0.028*	–	–	–	–
ΔSFT	ΔPHA	55	1.036	0.419	–	–	–	–
ΔSFT	ΔWBC	51	2.294	0.044*	–	–	–	–
ΔALB	ΔIgG	39	2.342	0.046*	58	35	15.740	0.027*
ΔALB	ΔPHA	42	0.912	0.509	42	27	7.594	0.369
ΔALB	ΔWBC	39	0.603	0.749	61	36	5.952	0.545

‘Δ’ denotes ‘change in’, ‘MLR’ mass per unit length (kg in pups; Ln (kg) in juveniles), ‘SFT’ skinfold thickness (cm), ‘ALB’ albumin concentration (relative peak intensity), ‘IgG’ total immunoglobulin G concentration (mg mL^−1^), ‘WBC’ total leukocyte concentration (10^9^ L^−1^) and ‘PHA’ response to phytohemagglutinin (mm).

**Table 2:** The effects of changes in immune measures on changes in body condition for models of relationships that explained significantly more variation than equivalent null models (Table 1); see Tables S2–3 for full model details.

	Condition Variable	Colony	Immune Variable	N	Slope	SE	*t*	*p*
Pups	ΔMLR (kg)	HIC	ΔIgG (mg/ml)	27	−0.0511	0.0214	−2.385	0.025*
		CC (Females)	ΔIgG (mg/ml)	24	0.1134	0.0353	3.208	0.004**
	ΔMLR (kg)	HIC	ΔWBC (10^9^/l)	25	0.1604	0.1458	1.100	0.283
		CC	ΔWBC (10^9^/l)	26	0.3613	0.1228	2.941	0.007**
	ΔSFT (cm)	HIC	ΔIgG (mg/ml)	24	−0.0042	0.0019	−2.262	0.034*
		CC	ΔIgG (mg/ml)	23	0.0100	0.0035	2.855	0.010*
	ΔSFT (cm)	HIC	ΔWBC (10^9^/l)	25	0.0231	0.0124	1.864	0.076
		CC	ΔWBC (10^9^/l)	26	0.0266	0.0175	1.514	0.144
	ΔALB (relative PI)	HIC	ΔIgG (mg/ml)	22	−0.0018	0.0008	−2.235	0.038*
		CC	ΔIgG (mg/ml)	17	−0.0003	0.002	−0.176	0.863
Juveniles	ΔALB (relative PI)	HIC (Males)	ΔIgG (mg/ml)	30, 17	−0.0073	0.0033	−2.244	0.034*
		CC	ΔIgG (mg/ml)	28, 18	0.0003	0.0032	0.101	0.920

‘Δ’ denotes ‘change in’, ‘MLR’ mass per unit length (kg), ‘SFT’ skinfold thickness (cm), ‘ALB’ albumin concentration (relative peak intensity), ‘IgG’ total immunoglobulin G concentration (mg mL ^−1^), ‘WBC’ total leukocyte concentration (10^9^ L^−1^) and ‘PHA’ response to phytohemagglutinin (mm). Juvenile sample sizes are shown as the total number of data points followed by the number of individuals.

## Discussion

The findings of this study have two important aspects. First, a subset of the observed results is consistent with a negative effect of changes in IgG concentration on changes in physiological condition. Although the correlative nature of the evidence precludes inference of the direction of the effect or causation as its driver, such evidence from the wild is rare, and such systems are rarely manipulable. Second, this statistical effect was only evident in the colony where sea lions were exposed to anthropogenic environmental impacts [28], [35]–[36] and the presence of domestic animals [26]–[27], [56]. Together our results suggest that human influence may have an indirect negative effect on Galapagos sea lion fitness through effects on immunity and body condition.

In pups from the control colony we observed the positive relationship between changes in immune measures and condition that would be expected under condition-dependent investment in immunity [57]. This suggests that under circumstances not strongly influenced by humans, Galapagos sea lions invest in immunity and growth according to the resources they have available. Those that have better access to resources, because they were born to more experienced mothers, for example, may invest more in both immunity and condition, which would result in the positive correlation that we observed and that would be expected under phenotypic correlation [57]. It should be noted, though, that this proposed explanation runs contrary to the direction of causation implicitly hypothesised by the set-up of the statistical models. In other words, the fitting of condition as the response variable and immune activity as the explanatory variable presupposes that changes in the former drive changes in the latter. This, of course, will not always be the case, but was the most appropriate of the two options of linear and mixed effects model fitting (which allowed for variation in other factors to be taken into account) that were available to us, because this was the hypothesis we were most interested in testing.

The negative relationships between change in IgG concentration and all three measures of body condition in the human-impacted colony may have been caused by a trade-off between the energy and resources consumed by IgG production and those available for growth and development [58]. The establishment of circulating protective antibody in young mammals is driven by response to the post-natal antigenic environment [40], [59]. IgG may have increased in concentration more in pups from the human-impacted colony than the control colony because they experienced a richer post-natal antigenic environment. The reason that these larger increases in IgG concentration in the human-impacted colony, but not those smaller increases observed in the control colony, were associated with a loss of body condition, may be because Galapagos sea lion immune system ontogeny has adapted in an environment free from human influence. In this case, developing Galapagos sea lion immune systems would have been selected to respond to their antigenic environment with sensitivity appropriate to human-free conditions. This would confer an advantage in the control colony, where present conditions are relatively unchanged from historical ones. However, such sensitivity may be disadvantageous in the newly antigen-rich environment of the human-impacted colony, where sea lions come into close contact with domestic animals, where the bay in which the sea lions live is home to more than two hundred vessels and is contaminated with faecal coliform bacteria by sewage from the town water system [28], [35]–[36].

Although less likely given the young age of the pups sampled in this study, it is also possible that pathogens drove the negative relationships observed in the human-impacted colony. IgG may have been produced in response to infection, so pups that experienced the greatest increases in IgG concentration may have decreased most in body condition due to the direct costs of infection, rather than the correlated costs of antibody production [60]. Such an effect of infection may not have been evident in total leukocyte and PHA data, because total leukocyte concentration and PHA response as measures of immune variation are more ephemeral and less cumulative than IgG concentration. None of the animals included in this study showed any outward signs of sickness, but we were unable to measure pathogen burden or clinical indicators of disease, despite screening faecal samples and blood smears for signs of infection. We were therefore unable to test whether there were higher levels of infection in the human-impacted colony compared with the control, or whether pathogen burden was positively correlated with IgG concentration. In addition, although they are unlikely to be principal drivers of the patterns of immune activity reported from these colonies [38], the possible influence of other factors such as stress, pollution, inbreeding and population density, which are discussed in depth in Brock et al. 2012 [38], should also be borne in mind.

If causality underlies the negative correlation between the changes in immune activity and changes in condition in the human-impacted colony, and the former drives the latter, our findings have interesting implications for Galapagos sea lion life history and disease risk, regardless of the directness of the causal linkage between the two. If the relatively high changes in IgG concentration in the human-impacted colony were caused by immune response to infection and were protective, then any down-regulation of the antibody response would increase the risk of disease to individuals and the population. Food shortages are known to down-regulate antibody-mediated immunity [61], so the rapid decreases in food availability driven by unpredictable environmental variation to which the Galapagos sea lion is exposed [24]–[25] could increase disease risk. On the other hand, if the relatively high changes in IgG concentration of the human-impacted colony were due to stimulation by antigens and microorganisms that are not typically virulent to sea lions (e.g. bacteria in human effluent; [28], [35]–[36]) during early immune system development and the sensitivity to this stimulation were not modulated in response to energy availability, sea lions of the human-impacted colony would be at greater risk from climate-driven decreases in food supply, as antigenic pressure would drain energy and resources through their immune systems. Under this scenario, sea lions in the human-impacted colony would be at greater risk of death from starvation and adults would have less energy available for reproduction [62]. If such effects were sustained, they could undermine colony stability and contribute to a population decline.

Even if the direction of causation that underlies the negative correlation in the human-impacted colony were reversed, the above implications would still apply. Regardless of whether an investment in immune activity necessitated decreased investment in body condition or *vice versa*, it is the negativity of this relationship rather than the direction of its underlying causation that determines its implications; specifically, the consistency of the observed negative correlation with a trade-off between investment in resistance to infection and starvation.

We were principally interested in the relationship between immune measures and condition and how this varied between colonies, but it was also important to consider the role that sex could have played in shaping or obscuring any such patterns. Sex differences in the way condition changes with age can arise through sex-specific modes of growth, development and maternal investment [46], [52]. In addition, changes in food availability have been shown to have different effects on male and female immune responsiveness [63], and immune challenge has been shown to differentially affect male and female body condition [64]. It is noteworthy that the positive relationship between change in IgG concentration and change in mass per unit length in the control colony was only evident in females. This may be because males and females grow in different ways: the sexes may regulate their subcutaneous fat stores in a similarly condition-dependent manner, but perhaps only females modulate their relative investments in skeletal and tissue growth in this way. The fact that the negative relationship between change in IgG concentration and change in serum albumin concentration in juveniles was only observed in males is curious. Given that there were neither sex differences in change in albumin concentration nor in change in IgG concentration in juveniles [38], this result suggests that the physiological correlates of changes in IgG concentration in juvenile males are fundamentally different from those in females.

The detection of life history trade-offs in the wild is complicated by variation that is often difficult to account for [65], [42], especially when immune responses and disease processes are involved [21]. In this study, by taking advantage of the unique ecology of the Galapagos sea lion, we have shown that ecological circumstances can modulate the relationship between immunity and condition in the wild. Although statistical replication beyond two colonies would not be possible, as the situation of the sea lion colony in the town of Puerto Bazquerizo Moreno is unique, our results have important implications for Galapagos sea lion conservation and suggest that subtle anthropogenic impacts that are difficult to study in the wild may be more common than we currently appreciate. Globally, as human pressure on wild systems increases, it becomes ever more important to understand these effects and their potential contribution to population declines through interactions with resource availability and the phenotypic plasticity of traits that have evolved in environments without ubiquitous human impacts.

## Supporting Information

Table S1 **Average changes in immune and condition measures by age class and colony.** ‘Δ’ denotes ‘change in’, ‘MLR’ mass per unit length (kg; Ln (kg) in juveniles), ‘SFT’ skinfold thickness (cm), ‘ALB’ albumin concentration (relative peak intensity), ‘IgG’ total immunoglobulin G concentration (mg mL ^−1^), ‘WBC’ total leukocyte concentration (10^9^ L^−1^) and ‘PHA’ response to phytohemagglutinin (mm). Note that juvenile sample sizes refer to the number of samples rather than the number of individuals.
(DOCX)

Table S2 **Full models of the five selected relationships between change in an immune measure and change in a condition variable in pups.** The effects of sex are reported as contrasts and females were used as the reference sex.
(DOCX)

Table S3 **Full models of the selected relationship between change in IgG concentration and change in albumin concentration in juveniles.** The effects of sex are reported as contrasts and males were used as the reference sex.
(DOCX)
