# Supplementary material for: Immune Activity, Body Condition and Human-Associated Environmental Impacts in a Wild Marine Mammal
Source: PLoS One. 2013 Jun 28;8(6):e67132. doi: 10.1371/journal.pone.0067132 (PMC3695956; doi:10.1371/journal.pone.0067132)
Supplement: Table S1 — Average changes in immune and condition measures by age class and colony. ‘Δ’ denotes ‘change in’, ‘MLR’ mass per unit length (kg; Ln (kg) in juveniles), ‘SFT’ skinfold thickness (cm), ‘ALB’ albumin concentration (relative peak intensity), ‘IgG’ total immunoglobulin G concentration (mg mL −1), ‘WBC’ total leukocyte concentration (109 L−1) and ‘PHA’ response to phytohemagglutinin (mm). Note that juvenile sample sizes refer to the number of samples rather than the number of individuals. (DOCX) [file pone.0067132.s001.docx]

Table S1. Average changes in immune and condition measures by age class and colony. ‘Δ’ denotes ‘change in’, ‘MLR’ mass per unit length (kg; Ln (kg) in juveniles), ‘SFT’ skinfold thickness (cm), ‘ALB’ albumin concentration (relative peak intensity), ‘IgG’ total immunoglobulin G concentration (mg mL ^-1^), ‘WBC’ total leukocyte concentration (10^9^ L^-1^) and ‘PHA’ response to phytohemagglutinin (mm). Note that juvenile sample sizes refer to the number of samples rather than the number of individuals.

|  | Mean change in pups (± SD, N) | | Mean change in juveniles (± SD, N) | |
| --- | --- | --- | --- | --- |
|  | Control | Human-impacted | Control | Human-impacted |
| ΔMLR (kg; Ln kg) | 0.90 (± 1.52, 27) | 0.05 (± 1.54, 28) | -0.004 (± 0.15, 38) | 0.03 (± 0.13, 35) |
| ΔSFT (cm) | 0.04 (± 0.20, 27) | 0.06 (± 0.14, 28) | - | - |
| ΔALB (relative PI) | -0.02 (± 0.08, 18) | -0.002 (± 0.05, 24) | -0.02 (± 0.07, 29) | 0.009 (± 0.09, 31) |
| ΔIgG (mg mL ^-1^) | 9.42 (± 10.85, 24) | 16.27 (± 12.95, 27) | 1.71 (± 5.21, 38) | -0.17 (± 7.19, 35) |
| ΔPHA (mm) | -0.11 (± 0.87, 27) | 0.36 (± 0.62, 28) | 0.06 (± 0.49, 29) | -0.33 (± 1.40, 20) |
| ΔWBC (10^9^ L^-1^) | -1.60 (± 2.60, 26) | -1.68 (± 2.77, 26) | -0.34 (± 2.45, 38) | 0.08 (± 3.00, 35) |
