# Supplementary material for: Immune Activity, Body Condition and Human-Associated Environmental Impacts in a Wild Marine Mammal
Source: PLoS One. 2013 Jun 28;8(6):e67132. doi: 10.1371/journal.pone.0067132 (PMC3695956; doi:10.1371/journal.pone.0067132)
Supplement: Table S2 — Full models of the five selected relationships between change in an immune measure and change in a condition variable in pups. The effects of sex are reported as contrasts and females were used as the reference sex. (DOCX) [file pone.0067132.s002.docx]

Table S2. Full models of the five selected relationships between change in an immune measure and change in a condition variable in pups. The effects of sex are reported as contrasts and females were used as the reference sex.

|  | Colony | N | R^2^ | Model term | Estimate | SE | *t* | *p* |
| --- | --- | --- | --- | --- | --- | --- | --- | --- |
| ΔMLR ~ ΔIgG + Sex | HIC | 27 | 0.27 | Intercept | 0.2315 | 0.4749 | 0.488 | 0.630 |
|  |  |  |  | ΔIgG | -0.0511 | 0.0214 | -2.385 | 0.025 * |
|  |  |  |  | Sex (male) | 1.2590 | 0.5453 | 2.309 | 0.030 * |
|  |  |  |  |  |  |  |  |  |
| ΔMLR ~ ΔIgG * Sex | CC | 24 | 0.34 | Intercept | 0.0029 | 0.4545 | 0.007 | 0.994 |
|  |  |  |  | ΔIgG | 0.1134 | 0.0353 | 3.208 | 0.004 ** |
|  |  |  |  | Sex (male) | 1.0054 | 0.7334 | 1.371 | 0.186 |
|  |  |  |  | ΔIgG * Sex (male) | -0.1291 | 0.0511 | -2.528 | 0.020 * |
|  |  |  |  |  |  |  |  |  |
| ΔMLR ~ ΔWBC + Sex | HIC | 25 | 0.18 | Intercept | -0.3067 | 0.4936 | -0.621 | 0.540 |
|  |  |  |  | ΔWBC | 0.1604 | 0.1458 | 1.100 | 0.283 |
|  |  |  |  | Sex (male) | 1.1441 | 0.6159 | 1.858 | 0.077 |
|  |  |  |  |  |  |  |  |  |
| ΔMLR ~ ΔWBC + Sex | CC | 26 | 0.27 | Intercept | 0.9027 | 0.3686 | 2.449 | 0.022 * |
|  |  |  |  | ΔWBC | 0.3613 | 0.1228 | 2.941 | 0.007 ** |
|  |  |  |  | Sex (male) | 1.2114 | 0.6292 | 1.925 | 0.067 |
|  |  |  |  |  |  |  |  |  |
| ΔSFT ~ ΔIgG + Sex | HIC | 24 | 0.20 | Intercept | 0.1207 | 0.0449 | 2.688 | 0.013 * |
|  |  |  |  | ΔIgG | -0.0042 | 0.0019 | -2.262 | 0.034 * |
|  |  |  |  | Sex (male) | 0.0314 | 0.0462 | 0.681 | 0.503 |
|  |  |  |  |  |  |  |  |  |
| ΔSFT ~ ΔIgG + Sex | CC | 23 | 0.34 | Intercept | -0.0972 | 0.0552 | -1.759 | 0.093 |
|  |  |  |  | ΔIgG | 0.0100 | 0.0035 | 2.855 | 0.010 ** |
|  |  |  |  | Sex (male) | 0.0895 | 0.0735 | 1.218 | 0.237 |
|  |  |  |  |  |  |  |  |  |
| ΔSFT ~ ΔWBC + Sex | HIC | 25 | 0.26 | Intercept | 0.0320 | 0.0419 | 0.764 | 0.453 |
|  |  |  |  | ΔWBC | 0.0231 | 0.0124 | 1.864 | 0.076 |
|  |  |  |  | Sex (male) | 0.1003 | 0.0523 | 1.919 | 0.068 |
|  |  |  |  |  |  |  |  |  |
| ΔSFT ~ ΔWBC + Sex | CC | 26 | 0.15 | Intercept | 0.0097 | 0.0526 | 0.185 | 0.854 |
|  |  |  |  | ΔWBC | 0.0266 | 0.0175 | 1.514 | 0.144 |
|  |  |  |  | Sex (male) | 0.1738 | 0.0899 | 1.933 | 0.066 |
|  |  |  |  |  |  |  |  |  |
| ΔALB ~ ΔIgG + Sex | HIC | 22 | 0.30 | Intercept | 0.0481 | 0.0188 | 2.559 | 0.019 * |
|  |  |  |  | ΔIgG | -0.0018 | 0.0008 | -2.235 | 0.038 * |
|  |  |  |  | Sex (male) | -0.0252 | 0.0193 | -1.304 | 0.208 |
|  |  |  |  |  |  |  |  |  |
| ΔALB ~ ΔIgG + Sex | CC | 17 | 0.01 | Intercept | -0.0111 | 0.0289 | -0.385 | 0.706 |
|  |  |  |  | ΔIgG | -0.0003 | 0.0018 | -0.176 | 0.863 |
|  |  |  |  | Sex (male) | -0.0119 | 0.0419 | -0.283 | 0.781 |
