# Supplementary material for: Immune Activity, Body Condition and Human-Associated Environmental Impacts in a Wild Marine Mammal
Source: PLoS One. 2013 Jun 28;8(6):e67132. doi: 10.1371/journal.pone.0067132 (PMC3695956; doi:10.1371/journal.pone.0067132)
Supplement: Table S3 — Full models of the selected relationship between change in IgG concentration and change in albumin concentration in juveniles. The effects of sex are reported as contrasts and males were used as the reference sex. (DOCX) [file pone.0067132.s003.docx]

Table S3. Full models of the selected relationship between change in IgG concentration and change in albumin concentration in juveniles. The effects of sex are reported as contrasts and males were used as the reference sex.

| Table S2. | Colony | N_total_ | N_inidividuals_ | Model term | Estimate | SE | *t* | *p* |
| --- | --- | --- | --- | --- | --- | --- | --- | --- |
| ΔALB ~ ΔIgG * Sex | HIC | 30 | 17 | Intercept | 0.0086 | 0.0255 | 0.337 | 0.739 |
| Random, time-point SD = 0.028 | | |  | ΔIgG | -0.0073 | 0.0033 | -2.244 | 0.034 * |
| Random, individual SD = 0.059 | | |  | Sex (female) | 0.0124 | 0.0251 | 0.494 | 0.626 |
| Random, residual SD = 0.022 | | |  | ΔIgG * Sex (female) | 0.0118 | 0.0042 | 2.819 | 0.010 * |
|  |  |  |  |  |  |  |  |  |
| ΔALB ~ ΔIgG * Sex | CC | 28 | 18 | Intercept | -0.0201 | 0.0193 | -1.037 | 0.310 |
| Random, time-point SD < 0.0001 | | |  | ΔIgG | 0.0003 | 0.0032 | 0.101 | 0.920 |
| Random, individual SD = 0.0600 | | |  | Sex (female) | -0.0016 | 0.0250 | -0.064 | 0.950 |
| Random, residual SD = 0.0003 | | |  |  |  |  |  |  |
